# SETD6 regulates NF-κB signaling in urothelial cell survival: Implications for bladder cancer

**DOI:** 10.18632/oncotarget.14750

**Published:** 2017-01-19

**Authors:** Neelam Mukherjee, Eduardo Cardenas, Roble Bedolla, Rita Ghosh

**Affiliations:** ^1^ Departments of Urology, School of Medicine, University of Texas Health Science Center at San Antonio, San Antonio, TX 78229, USA; ^2^ Departments of Pharmacology, School of Medicine, University of Texas Health Science Center at San Antonio, San Antonio, TX 78229, USA; ^3^ Departments of Molecular Medicine and School of Medicine, University of Texas Health Science Center at San Antonio, San Antonio, TX 78229, USA; ^4^ Departments of Cancer Therapy and Research Center, School of Medicine, University of Texas Health Science Center at San Antonio, San Antonio, TX 78229, USA

**Keywords:** SETD6, methyltransferase, NF-κB, bladder cancer, signaling

## Abstract

Non-muscle invasive bladder cancer has a high recurrence rate of 45-70%, progressing to muscle invasive disease in about 15% of those patients over a 5-year period. Administration of the mycobacterium, Bacillus Calmette-Guerin (BCG) that induces local inflammation resulting in tumor remission in responsive patients is frequently used for treatment. BCG-treated patients with NF-κB del/del genotype have an increased risk of recurrence suggesting an important role of NF-κB in bladder cancer. Since protein methyltransferases play critical roles in modulating chromatin structure and gene expression, we screened a focused array of epigenetic modification genes to identify differential expression between normal urothelial and bladder cancer cells. We found and validated high expression of the SET-domain-containing protein methyltransferase, SETD6. SETD6 monomethylates NF-κB-p65 at lysine 310. Our results show that primary urothelial cells and normal bladder tissue have nearly undetectable message and protein level of SETD6 that increases in transformed urothelial cells and is further increased in bladder cancer cells and tissues. Overexpression of SETD6 in transformed urothelial cells increased cell survival and colony formation while knockdown in cancer cells decreased both parameters. Luciferase reporter assays showed that SETD6 induced the canonical NF-κB signaling pathway. Further, the use of catalytic SETD6 and IκBα mutant shows that SETD6 positively regulates survival by affecting p65 message, protein level and its function as determined by increased expression of NF-κB target genes. Our findings suggest that SETD6 plays an important role in NF-κB regulation and may have an important role in NF-κB-mediated local inflammatory response following BCG treatment.

## INTRODUCTION

Bladder cancer is the second most common urologic malignancy, accounting for about 74,000 newly diagnosed cases and over 16,000 deaths in the United States in 2015 [1]. The first line of therapy for non-invasive bladder cancer is transurethral resection of bladder tumor (TURBT). Following TURBT different forms of adjuvant therapy are used to prevent recurrence and progression of bladder cancer. The most frequently used therapy is the administration of Bacillus Calmette-Guerin (BCG), a mycobacterium that is directly instilled into the bladder [2]. Despite these strategies recurrence is as high as 70% with about 15% of recurrent cancers progressing to muscle invasive disease. The mechanism through which BCG functions is still not clearly understood. Although local inflammation is considered to be a positive sign for BCG therapy [3], chronic inflammation and modulation of chemokines and cytokines, regulated by transcription factors, has been established as a major player in cancer-related inflammation [4, 5]. As a result inflammation can be considered as an enabling characteristic for progression of carcinogenesis as well as necessary for response to therapeutic modalities [5]. Interestingly, increased expression of NF-κB1 is associated with BCG treatment, reflecting a role for NF-κB in BCG-mediated immune response [6]. BCG-treated patients with NF-κB del/del genotype were reported to have a 2.5-fold increased risk of recurrence compared to ins/ins genotype [7].

Nuclear expression of the p65 subunit of NF-κB increases with increasing tumor grade and T-category in bladder cancer [8]. NF-κB also has been shown to play a role in the pathogenesis of transitional cell carcinoma (TCC) [9]. Notably, polymorphisms of the NF-κB promoter have also been detected in bladder cancer [10]. Specifically, an insertion/deletion ATTG functional polymorphism at –94 has been specifically linked with bladder cancer recurrence [10]. Like many regulatory proteins, NF-κB undergoes post-translational modifications to affect its downstream signaling [11]. For example methylation of NF-κB at K218 and K221 increases the transcriptional activity of NF-κB at its target genes [12]. Further, arginine methylation mediated by arginine methyltransferases, such as PRMT1 and PRMT2, regulate NF-κB-dependent transcription [13]. Further, the methyltransferase, SETD6 methylates p65 at lysine 310 and modulates its transactivation properties [14].

SETD6 is mainly composed of the helical structures of i-SET (an insertion of about 125 amino acids in the middle of the SET domain) and the C-terminal domain that is made up of mainly α-helices and a few β-strands [15]. The role of SETD6 in cancer is poorly defined. Transcription profile analysis shows upregulation of SETD6 in diffuse gastric adenocarcinoma, adenocarcinoma of the colon, rectum, cecum, and rectal mucinous adenocarcinoma compared to their respective normal tissues [16]. However, in liquid cancers such as B cell childhood acute lymphoblastic leukemia and classical Hodgkin's lymphoma, SETD6 expression was found to be downregulated [17]. Examination of bladder cancer gene expression datasets failed to provide conclusive results regarding SETD6 expression in bladder cancer [18–23]. To the best of our knowledge, expression and function of SETD6 in bladder cancer remains unaddressed.

In this study, we identified SETD6 as a significantly upregulated gene in bladder cancer cells and tissues. We also delineated for the first time the function of SETD6 as a novel regulator of NF-κB levels and signaling in bladder carcinogenesis. This study shows that SETD6 induces NF-κB signaling by increasing p65 level and function and challenges the currently known role of SETD6 in post-translational modification of NF-κB.

## RESULTS

### Higher level of SETD6 is associated with bladder cancer

**Figure 1:**
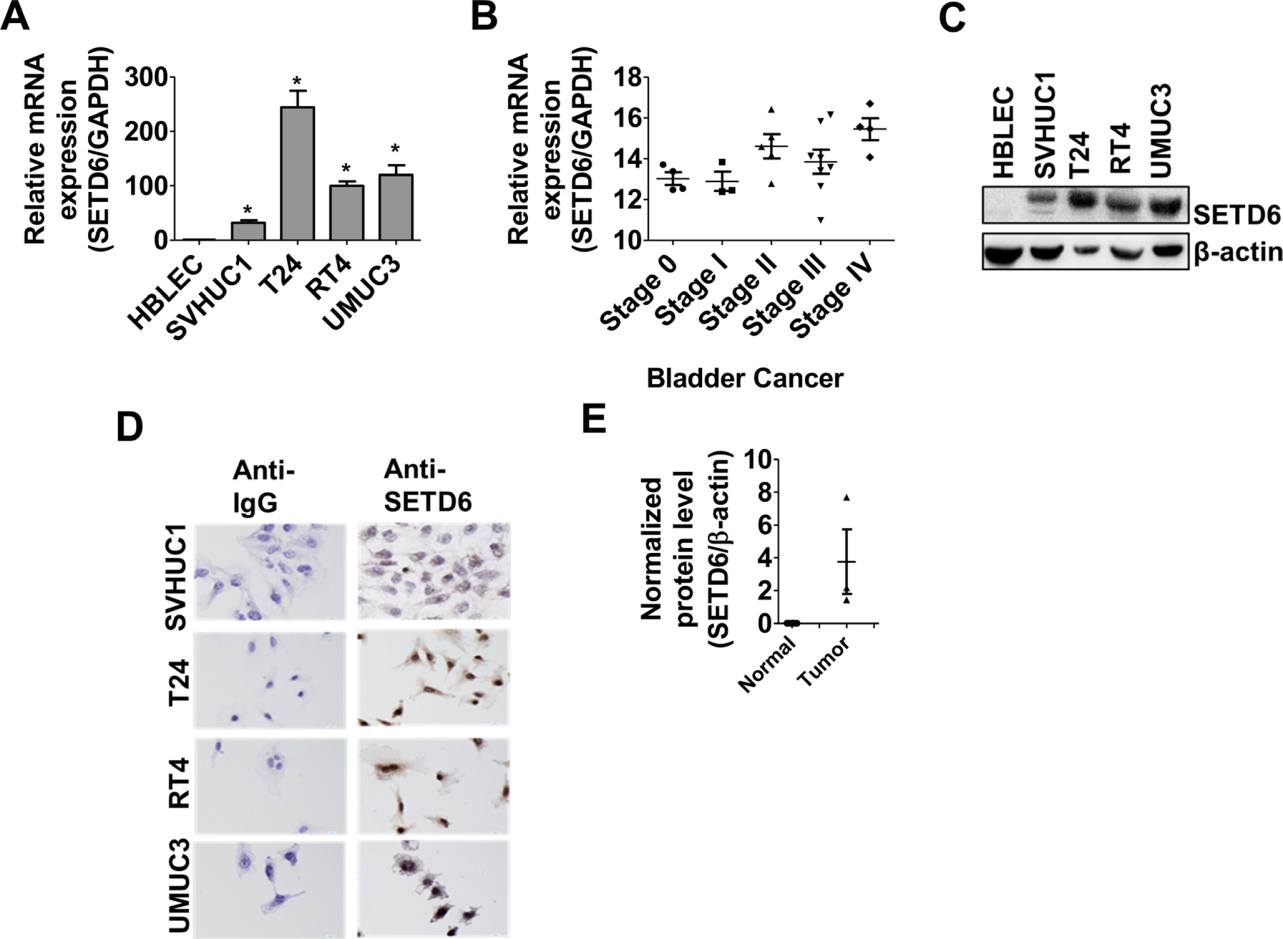
Upregulation of SETD6 in bladder cancer (**A**) Validation of relative SETD6 expression in different bladder cells was carried out using qPCR. *p ≤* 0.05 were considered to be statistically significant (*). The samples were measured in triplicates and the experiment was repeated 3 times. (**B**) cDNA array was performed with bladder cancer tissues (*n* = 24) representing different stages and SETD6 expression was analyzed by qPCR. (**C**) Basal level of SETD6 protein was detected using anti-SETD6 antibody by western blotting. (**D**) Immunocytochemistry in different bladder cancer cell lines cells showing cytoplasmic and nuclear SETD6. (**E**) SETD6 protein was detected using anti-SETD6 antibody by western blot in bladder cancer tissues (*n* = 9) compared to non-cancerous bladder tissues.

A focused chromatin array was used to identify differentially regulated chromatin-remodeling genes between the human bladder cancer cell line (T24) and primary urothelial cells (HBLEC). It led to the identification of differential SETD6 expression (Supplementary Figure 1A). Validation of SETD6 expression using real-time qPCR showed low message levels in transformed (SVHUC1) and normal cells (HBLEC) and about 100 fold or higher expression in bladder cancer cell lines (T24, RT4, UMUC3; Figure 1A). We also found upregulation of SETD6 expression in other cancer cell lines (melanoma, pancreas and prostate) albeit at much lower level compared to bladder cancer cells (Supplementary Figure 1B). Although not conclusive, analysis of Oncomine data indicates a trend (non-significant) towards increased SETD6 copy number, expression with increased stage of bladder cancer and data from the TCGA suggests that SETD6 copy number may be affected by smoking status [23]. Smoking is a major risk factor for bladder cancer. We used a human bladder tissue cDNA array that included samples from patients at different stages of bladder cancer (*n* = 24) to determine whether the cell line observations were of clinical significance. Message levels show that expression of SETD6 correlates with stage of bladder cancer, suggesting that SETD6 may have a role in bladder cancer progression (Figure 1B). To confirm the mRNA results, we used immunoblotting to determine SETD6 protein levels and found that SETD6 was minimally detectable in primary urothelial cells, higher in transformed bladder epithelial cells and > 50 fold higher level in bladder cancer cell lines compared with primary cells (Figure 1C and Supplementary Figure 2). Immunocytochemistry was used to determine SETD6 protein levels and it confirmed low basal level of SETD6 in the transformed cell line, SVHUC1, compared to bladder cancer cell lines (Figure 1D). SETD6 protein level was 4-6 fold higher in bladder cancer tissue samples compared with the normal urothelial tissue (*n* = 9; Figure 1E). These observations taken together suggest that SETD6 may be upregulated in bladder cancer to confer a survival advantage.

### SETD6 increases viability and proliferation of transformed bladder cells

**Figure 2:**
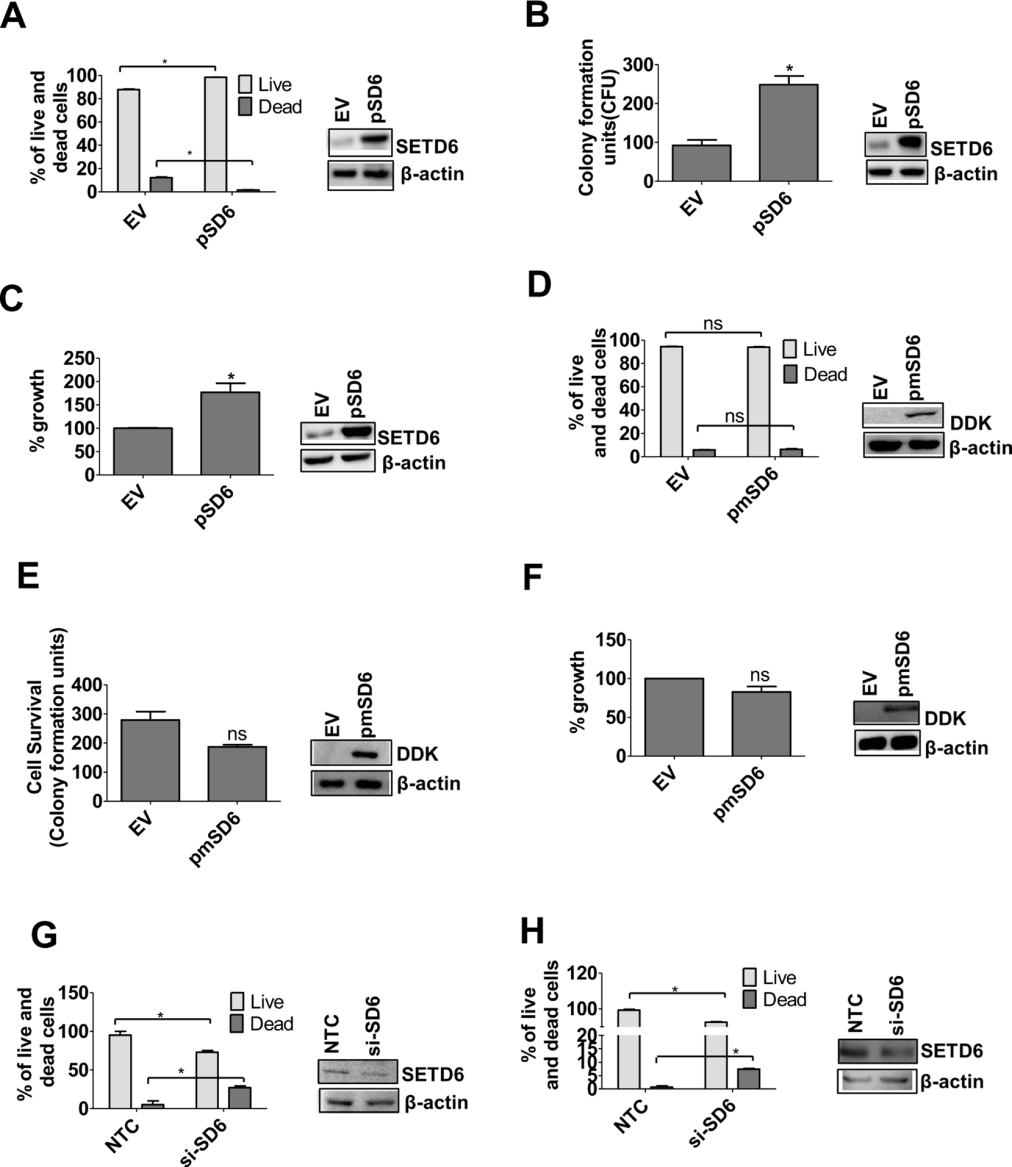
SETD6 positively regulates survival Wild type (wt) SETD6 was overexpressed in the SVHUC1 cell line. EV: pCAG; pSD6: pCAG-Flag SETD6. (**A**) Trypan blue and (**B**) crystal violet staining were used to determine cell survival. The samples were counted in duplicates and the experiments were repeated 2 times. *p ≤* 0.05 were considered to be statistically significant (*). (**C**) MTT assay was used to measure growth. The samples were measured in triplicates and the experiments were repeated 3 times. *p ≤* 0.05 were considered to be statistically significant (*). Mutant (mut) SETD6 Y285A was overexpressed in the SVHUC1cell line. EV: pCAG; pmSD6: pCAG-Flag mutant SETD6 (Y285A). (**D**) Trypan blue and (**E**) crystal violet staining were used to determine cell survival. The samples were counted in duplicates and the experiments were repeated 2 times. *p ≤* 0.05 were considered to be statistically significant (*). (**F**) MTT assay was used to measure growth. The samples were measured in triplicates and the experiments were repeated 2 times. *p ≤* 0.05 were considered to be statistically significant (*). SETD6 was knocked down in bladder cancer cell lines (**G**) in UMUC3 and (**H**) in RT4 and trypan blue assay was used to determine cell survival. NTC: non-targeting control siRNA; si-SD6: SETD6 siRNA. Samples were counted in duplicate and the experiments were repeated twice. *p ≤* 0.05 were considered to be statistically significant (*).

To determine the functional significance of SETD6 overexpression in bladder cancer cells, we tested the idea that SETD6 provides growth advantage to urothelial cells. Overexpression of wild type (wt) SETD6 in the transformed cell line, SVHUC1, (with low endogenous SETD6) significantly increased cell viability and decreased cell death (Figure 2A). We also determined the effect of SETD6 overexpression on colony formation and ability to proliferate. There was a significant increase in colony formation and percentage of proliferating cells upon SETD6 overexpression (Figure 2B and 2C). To determine whether the observed growth impetus requires the catalytic function of SETD6, we used a SETD6 catalytic mutant; Y285A. Our results show no significant change in viability, colony formation or proliferative ability in cells overexpressing the catalytic mutant SETD6 (Figure 2D–2F). These results show that the catalytic activity of SETD6 is necessary for its pro-survival function in transformed bladder cancer cells. To confirm the pro-survival function of SETD6 we used RNAi to knock down SETD6 in the bladder cancer cell lines, RT4 and UMUC3, (high endogenous levels of SETD6). In keeping with our observations above, SETD6 knockdown inhibited survival of both bladder cancer cell lines and increased the percentage of cells undergoing death (Figure 2G and 2H). These data together support our premise regarding the pro-survival role of SETD6 in bladder cancer.

### SETD6 stimulates NF-κB signaling

**Figure 3:**
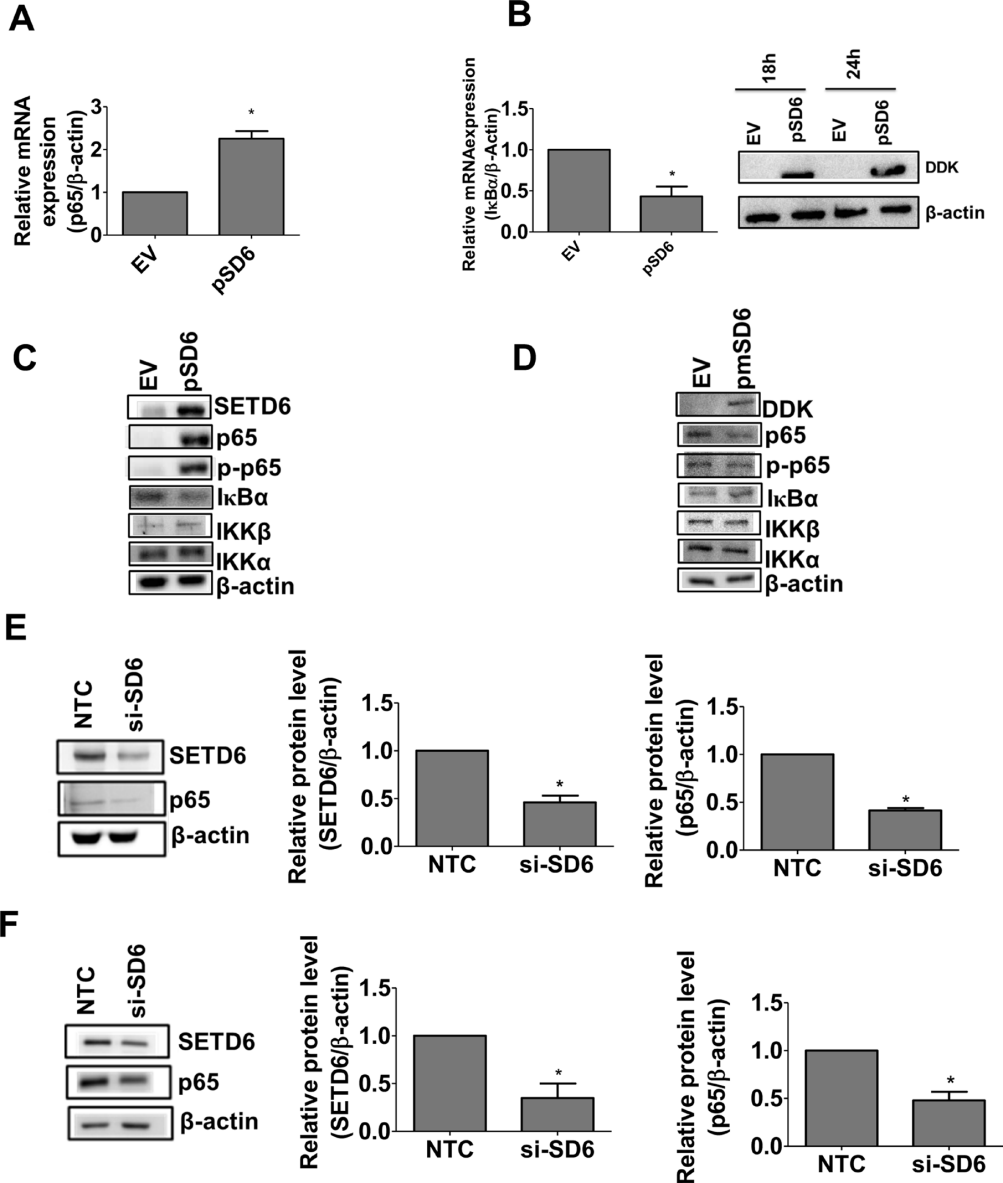
SETD6 induces canonical NF-κB signaling Effect of SETD6 overexpression on (**A**) p65 mRNA and on (**B**) IκBα mRNA (18 h post SETD6 overexpression) measured by RT-PCR. Samples were measured in triplicates and experiments were repeated 3 times. *p ≤* 0.05 were considered to be statistically significant (*). EV: (empty vector) pCAG; pSD6: pCAG-Flag SETD6. (**C**) Immunoblots to show the effect of wild type (wt) SETD6 overexpression on p65 using whole cell and nuclear extracts. EV (empty vector): pCAG; pSD6: pCAG-Flag SETD6. Other members of the NF-κB family were tested using whole cell extracts. (**D**) Effect of mutant (mut) SETD6 overexpression on p65 and other members of the NF-κB family as in panel C. EV (empty vector): pCAG; pmSD6: pCAG-Flag mutant SETD6 (Y285A). Effect of SETD6 knockdown on p65 in (**E**) UMUC3 and (**F**) RT4. *p ≤* 0.05 were considered to be statistically significant (*). Experiments were repeated twice. NTC: non-targeting control siRNA; si-SD6: SETD6 siRNA.

Given that the p65 subunit of NF-κB is a known target of SETD6 [14] we determined the effect of SETD6 overexpression on the regulation of the canonical NF-κB signaling pathway. Interestingly, SETD6 overexpression increased p65 and decreased IκBα mRNA levels (Figure 3A and 3B). These changes were consistent with increased p65 and decreased IκBα protein levels (Figure 3C and Supplementary Figure 3). SETD6 overexpression was also accompanied by significant increase in phosphorylated p65 (Ser 536), increased IKKβ and no change in IKKα (Figure 3C and Supplementary Figure 3). These observations suggest that SETD6 induces the canonical NF-κB signaling pathway. To confirm the involvement of SETD6 catalytic activity in NF-κB signaling, we overexpressed the catalytic SETD6 mutant. Our results show no effect of the mutant overexpression on NF-κB signaling (Figure 3D and Supplementary Figure 4). SETD6 knockdown in bladder cancer cell lines (UMUC3 and RT4) significantly decreased p65 levels (Figure 3E and 3F) suggesting that SETD6 regulates the protein level of p65. Overall these data show that SETD6 regulates the level and signaling of NF-κB for which the catalytic function of SETD6 may be necessary.

### SETD6 regulated p65 function supports survival of urothelial cells

**Figure 4:**
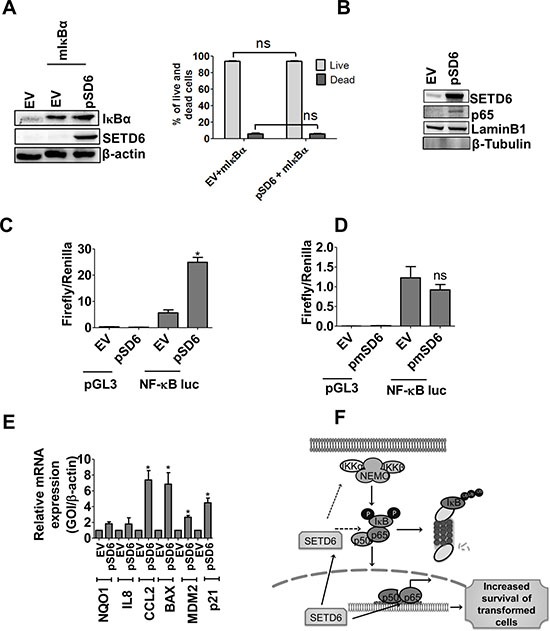
SETD6 mediated p65 level increases cell survival (**A**) Consequence of wild type (wt) SETD6 and mutant (mut) IκB overexpression on survival of SVHUC1 cells determined by trypan blue assay. The samples were measured in duplicates and experiments were repeated twice. *p ≤* 0.05 was considered to be statistically significant (*). Lane 1 EV (empty vector): pCAG+ pcDNA; Lane 2 EV (empty vector): pCAG; pSD6: pCAG-Flag SETD6; mIκBα: pcDNA-mutant IκBα (S32/36A). Effect of wt SETD6 overexpression on (**B**) nuclear level of p65 determined by western blotting and the effect on (**C**) transcription reporter activity of NF-κB measured by luciferase activity. The samples were measured in triplicates and experiments were repeated twice. *p ≤* 0.05 was considered to be statistically significant (*). (**D**) Effect of mut SETD6 overexpression on transcriptional activity of NF-κB measured by luciferase activity. Samples were measured in triplicates and experiments were repeated twice. *p ≤* 0.05 was considered to be statistically significant (*). (**E**) Effect of SETD6 overexpression on NF-κB target gene expression (24 h post SETD6 overexpression) was measured by RT-PCR. The samples were measured in triplicates and experiments were repeated twice. *p ≤* 0.05 was considered to be statistically significant (*). EV (empty vector): pCAG; pSD6: pCAG-Flag SETD6. (**F**) Model of the role of SETD6 in bladder cancer: SETD6 induces NF-κB signaling by increasing p65 level and its nuclear translocation resulting in increased survival of transformed bladder cells.

To determine whether SETD6 can affect NF-κB's function, we first determined whether co-expression of mutant IκBα (S32/36A) and SETD6 can attenuate the increased cell viability seen by SETD6 overexpression alone. The mutant IκBα construct cannot be phosphorylated; thus preventing its proteolytic degradation resulting in the inhibition of NF-κB activation. Our results show that SETD6 overexpression cannot overcome IκBα-mediated inhibition of NF-κB, and as such did not result in changes in survival of SVHUC1 cells (Figure 4A). Since activation of NF-κB occurs in response to various stimuli, we tested whether SETD6 overexpression serves as a stimulus to activate NF-κB. As shown in Figure 4B, the nuclear level of NF-κB was significantly increased in SETD6 overexpressing cells compared with vector transfected cells suggesting that SETD6 may serve as an inflammatory stimulus to activate p65. Luciferase reporter assay was used to determine whether SETD6 overexpression-mediated nuclear p65 was functionally active. Our results show a highly significant increase in reporter activity in cells overexpressing SETD6 (Figure 4C). However, overexpression of mutant SETD6 showed no significant change in NF-κB reporter activity suggesting the involvement of SETD6 catalytic activity in NF-κB function (Figure 4D). Further, increased transcriptional activity of NF-κB also significantly increased expression of several target genes tested (Figure 4E). Of these, NQO1, CCL2, and MDM2 are associated with increased risk or tumor promoting function in bladder cancer [24–27]. Upregulation of other NF-κB target genes (BAX, p21) that play critical roles in regulation of cell survival was also noted [28, 29]. However, not all target genes were induced which was not surprising since regulation by PKMTs is likely to be a fine-tuning mechanism for expression of subsets of genes based on the stimulus.

## DISCUSSION

Modulation of the epigenome, together with genetic changes, is important in cancer development and progression, including bladder cancer [30]. SUV39H1 was the first histone lysine methyltransferase [31] followed by several others including G9a/GLP [22, 32], EZH2[33], SET2 [34], SET7/9 [35], DOT1 [36]. Most protein methyltransferases contain a SET domain and are involved in adding methyl group(s) to lysine residues of histones and non-histone proteins. Methylation of p53, the most commonly mutated gene in invasive bladder cancer has been extensively studied and has been found to be methylated by SET7/9, SMYD2, SETD8, and G9a/GLP at K372, K370, K382, and K373, respectively [37]. Increased expression and protein levels of EZH2 has been reported in highly advanced bladder cancer specimens [38]. EZH2 silencing also resulted in significantly decreased cell proliferation and reduced G1 to S phase transition in bladder cancer cells [39]. PKMTs like DOT1L, SMYD2, JMJD2C have been reported in other cancers [40–42], but information on PKMTs are generally lacking in bladder cancer. We identified a novel PKMT, SETD6, to be highly upregulated in bladder cancer cell lines and tissues at the mRNA and protein level. Our data is in agreement with gene expression data in the Oncomine database. Information available in the TCGA database, showed no significant increase in copy number between normal bladder and cancer although the median copy number was higher in cancer compared with normal bladder tissue. The implication of this information as it relates to BCG treatment failure in patients is a topic of great interest in our laboratory. Further, overexpression of SETD6 increased survival and growth of transformed bladder cells whereas knockdown in cancer cells reversed its effects on growth and survival suggesting a pro-survival function of this PKMT in bladder cancer.

NF-κB is ubiquitously expressed in all cell types and tissues where it regulates gene expression by binding to promoters/enhancers of a host of genes. NF-κB activation occurs through either the classical or alternative pathways [43]. In the classical pathway, stimulation by pro-inflammatory cytokines activates the IKK complex resulting in the phosphorylation of IκB proteins on two N-terminal serine residues. In the alternative pathway, IKKα is phosphorylated by NF-κB inducing kinase (NIK), which phosphorylates p100 leading to polyubiquitination and degradation of the inhibitory molecules by the proteasome [44, 45]. The freed NF-κB dimers are translocated into the nucleus, where they transactivate target genes by binding to gene promoter/enhancer regions. Quantitative data of immunohistochemical levels of p65 expression in paraffin-embedded tissue from 116 bladder cancer patients showed concurrent cytoplasmic and nuclear expression, with only three patients being negative for p65 in a majority of the patients [8]. Univariate and multivariate analysis of superficial and muscle invasive carcinomas show nuclear p65 expression increases while cytosolic expression decreases with increasing tumor grade and T-category [8]. An *in situ* hybridization study of twenty TCC patients implicated NF-κB in the pathogenesis of TCC [9]. Although these lines of evidence point to an important role for NF-κB in bladder cancer; the regulation of NF-κB in urothelial cancer remains poorly defined.

SETD6 monomethylates p65 at Lys310 recruiting GLP (G9A-like protein) to the NF-κB target genes resulting in transcriptional modulation [14]. Our results showed that SETD6 exerted its pro-survival effect in bladder cancer by positively regulating the NF-κB signaling pathway (Figure 4F). We found that SETD6 induced the canonical signaling pathway and transactivation function of NF-κB resulting in the expression of downstream target genes. However, SETD6 had been shown previously to be involved in transcriptional repression of p65 in the osteosarcoma cell line U-2 OS cells [14]. Although the exact reason for this different response is unclear at this time, we speculate that the contextual difference in the role of NF-κB in transformed vs. cancer cells may determine the methylation ability of SETD6. It is also possible that SETD6 methylated p65 recruits other activating cofactors which in turn activates an otherwise repressive methylation mark in bladder cancer. It is also conceivable that SETD6-mediated inhibition of IκBα seen in our model is lost in the osteosarcoma cancer cell line.

A variety of adjuvant therapies of which administration of BCG is primarily used to prevent recurrence and progression of bladder cancer [2]. The association between NF-κB genotype and BCG treatment response underscores the importance of understanding the regulation of NF-κB in bladder cancer. Our results have implications for SETD6 mediated regulation of the inflammatory response that takes place locally at the site of BCG administration. Our work has critical implications for the use of NF-κB as a therapeutic target in bladder cancer by inhibiting its regulator, SETD6. Since NF-κB is an essential transcription factor that is required for normal cell physiology, complete inhibition of NF-κB cannot be accomplished. However, inhibiting SETD6 (epigenetic NF-κB modulator) that is upregulated in bladder cancer can be a potential therapeutic option to control the level of NF-κB in bladder cancer cells. Pro-inflammatory activity of NF-κB has been implicated in bladder cancer progression therefore, we speculate that inhibitors of SETD6 that can inhibit lysine methylation of p65 can be a potential therapeutic strategy for bladder cancer. Since the general mechanism of BCG response is through generation of an inflammatory response at the tumor site, we believe that pre-existing inflammation as a result of activated NF-κB hampers the ability of BCG to produce substantial inflammatory response to kill the tumor. Therefore, epigenetic inhibition of NF-κB activation can potentially stratify patients for BCG therapy. However, more work is needed to determine whether SETD6 can regulate NF-κB or its other inflammatory targets following BCG therapy.

Over the past decade investigation of PMT inhibitors have grown rapidly and inhibitors have been developed as potential therapeutic targets. This includes the first PKMT inhibitor chaetocin in 2005, the first PRMT inhibitor in 2004 and the clinical trials for the DOT1L inhibitor in 2012 and the EZH2 inhibitors in 2013 and 2014 [46]. We can exploit the existence of highly sophisticated chemical probes to further the screening and identification of selective inhibitors of SETD6. Further, in preliminary screening of natural products in our laboratory we identified palmatine as a potential SETD6 inhibitor, due to its ability to decrease the level of SETD6 protein. Palmatine, is a compound isolated from the extract of *Phellodendron amurense* bark [47]. Based on our data, we believe that SETD6 inhibition will have anti-cancer effects in bladder cancer. However, further experiments will be needed in animal models targeting SETD6 to substantiate its therapeutic capability.

## MATERIALS AND METHODS

### Cell culture

RT4, T24, SVHUC1, UMUC3 cells were obtained from American Type Culture Collection (ATCC). SVHUC1(transformed, SV40 immortalized) cells were cultivated in Ham's F12K (ATCC 30-2004, Manassas, VA. USA) media supplemented with 7% fetal bovine serum (FBS) (S11195, Atlanta Biologicals, Lawrenceville, GA. USA) and 1% penicillin-streptomycin (30-002-Cl) (Corning Cellgro^®^, Manassas, VA. USA). Bladder cancer cell lines, T24, and RT4 were cultured in McCoy's 5A (Corning), FBS (10%) and antibiotics. UMUC3 were cultured in MEM media (Corning), FBS (10%), sodium pyruvate (1%), non-essential amino acids (0.1%), sodium bicarbonate (2%). The human bladder epithelial cell lines HBLEC (FC-0040 Lot # 00997 & 01177) was purchased from Lifeline Cell Technology (Oceanside, CA) and were cultivated in ProstaLife™ basal media (LM-0017) supplemented with the ProstaLife™ LifeFactors kit (LS-1072) from LifeLine Cell Technology.

### Transfection

For overexpression experiments, cells were plated at a density of 100,000 cells per well in 6 well plates. Transient overexpression of wild type and mutant SETD6 were carried out the following day with pCAG Flag-SETD6 wt, pCAG Flag-SETD6 (Y285A) plasmids (gifts from Dr. Danny Levy, Ben Gurion University). SVHUC1 cells were also co-transfected with pCAG Flag-SETD6 wt and pcDNA-p65 wt (gifts from Dr. Danny Levy, Ben Gurion University) and pcDNA-IκB (S32/36A). All the transfections were carried out with 1 μg plasmid with corresponding empty vectors as controls. For the knockdown experiments, cells were plated at a density of 30, 000 cells per well in 12-well plates. RNAi-mediated knockdown of SETD6 was performed with 100 nM smart pool ON-TARGET plus SETD6 siRNA (Dharmacon, L-014486) and with non-targeted scrambled siRNA (Dharmacon, D-001810-01-05) according to the manufacturer's recommendations. Validation of overexpression and knockdown was determined by protein extraction and immunoblotting.

### cDNA array

cDNA array (Origene, TissueScan™ Cancer and Normal Tissue cDNA Arrays) was used to determine the level of SETD6 expression in different bladder tissue samples (*n* = 24). SETD6 primer (Integrated DNA Technologies, SETD6: forward- AGCTTTCAGGAACCACTGGAGGAA, reverse- ATGGCCTTTAGGAATGGGCTGAGT) was mixed with PCR master mix, equal volumes were added to each well of the same plate and subjected to real-time PCR cycling program. Quantitative PCR reactions were carried out using SYBR^®^Green PCR master mix (Applied Bio systems). Normalization was performed using GAPDH as a reference. qPCR reaction was executed in the ABI PRISM 7300 Real Time PCR system and Ct values were obtained from ABI PRISM 7300 Sequence Detector software by negative correlation with ROX dye serving as an internal reference control.

### Immunocytochemistry

Immunohistochemistry analysis was conducted according to established protocols [48]. Briefly, sections from paraffin-embedded cells were deparaffinized and rehydrated. For antigen retrieval, we used sodium citrate (pH 6) buffer with heat under pressure. The sections were blocked with 3% hydrogen peroxide TBS buffer and 10% bovine serum albumin TBS buffer. Antibodies used for staining were: SETD6 (1:200 dilution, cat #: HPA041481, Prestige Antibodies, Sigma-Aldrich). Negative control slides were incubated in Rabbit Universal Negative Control (DAKO Corp Carpinteria). Slides were developed using a polymer detection system (Biocare Medical Concord) and a DAB Chromogen System (DAKO).

### RNA extraction and quantitative real time PCR

Total RNA was extracted using TRIzol^®^ (Invitrogen, Carlsbad, CA, USA) and ethanol precipitation method. RNA measurements and quality were analyzed with the Nano Drop spectrophotometer (Thermo Scientific). The SuperScript^®^VILO™ cDNA synthesis kit (Life Technologies) was used to transcribe 2 μg of total RNA. The resulting cDNA was diluted 1:3 for subsequent quantitative PCR reactions using SYBR^®^Green PCR master mix (Applied Bio systems). Samples were measured in triplicates. Normalization was performed using β-actin (ACTB) as a reference. Values are expressed in terms of 2^–ΔΔC^t (^Δ^C_t_ = ^Δ^C_tsample_–^Δ^C_tcalibrator_) or 2^–ΔC^t (^Δ^Ct=C_tsample_–C_treference_). qPCR reaction was executed in the ABI PRISM 7300 Real Time PCR system and Ct values were obtained from ABI PRISM 7300 Sequence Detector software by negative correlation with ROX dye serving as an internal reference control. The following primers (Integrated DNA Technologies) were used at 5 μM for quantitative PCR: *SETD6*: forward-AGCTTTCAGGAACCACTGGAGGAA, reverse-ATGGCCTTTAGGAATGGGCTGAGT, p65:forward-AGAGGAGCACAGATACCACCAAGA, reverse-AGAGCTCAGCCTCATAGAAGCCAT, NQO1: forward-AAGGATGGAAGAAACGCCTGGAGA, reverse-GGCCCACAGAAAGGCCAAATTTCT, CCL2: forwardCAGCAAGTGTCCCAAAGAAGCTGT, reverse- TGGAATCCTGAACCCACTTCTGCT, reverse- IL8: forward-AGAAACCACCGGAAGGAACCATCT, reverse-AGAGCTGCAGAAATCAGGAAGGCT, p21: forward-CAGTGGGAATAGAGGTGATATTG, reverse- AGATCAGGAGGATGACATTAATAC, BAX: forward- CCCTGCCCGAAACTTCTAAA, reverse-CCAATGAGC ATCTCCCGATAA, MDM2:forward-GTTAAGTCCTGAC TTGTCTCCA, reverse-CTTACCTGGATCAGCAGAGAA A, IκBα:forward: AGGATGAGCTGCCCTATGATGA, reverse primer- TGCCACTTTCCACTTATAATGTCAGA.

### Cell viability assay

Trypan Blue assay was used. Cells were plated and desired transfection was carried out. 2 or 3 days after transfection, cells were trypsinized and then spun down and resuspended in 50 μl of PBS. 10 ul of the sample was mixed with 10 ul of trypan blue and mixed thoroughly. The mixed samples were immediately counted on a hemocytometer. Live cells appeared clear and dead cells stained blue. 2 replicates were used for each sample.

### Survival assay

Cells were plated at a density of 100,000 cells per well in 6 well plates and the desired transfection was carried out. Cells were trypsinized and re-plated at a density of 1000 cells/well 24 hours after the initial transfection. Cells were then allowed to grow for 5 days and crystal violet staining was performed and the number of colonies was counted.

### MTT assay

Cell Titer proliferation assay (Promega Corporation, Madison, WI) was used to determine the percentage of proliferating cells. Cells were plated in 6 well plates and desired transfection was carried out. 2 days after transfection cells were sub cultured and plated in 96 well plates at 5000 cells/well in triplicate. Next day, 15 μl of proliferation dye was added and incubated at 37°C for 4 hr. 100 μl of stop solution was added to the wells and incubated at 37°C overnight. The plate was scanned in the plate reader at 570 nm & 650 nm (as a reference wavelength) in a SpectraMax plate reader (Molecular Devices). Samples were measured in triplicates.

### Protein extraction and immunoblotting

Flash frozen human tissue (100-200 mg) (obtained from Dr.Robert Svatek, UTHSCSA, San Antonio) (*n* = 9) was first put in a mortar-pestle previously cleaned with pre-chilled PBS. Liquid nitrogen was carefully added and allowed to evaporate making sure that the time did not exceed 2 minutes. RIPA buffer containing protease and phosphatase inhibitor cocktail (1%) and DTT (5%) was added and tissue was homogenized. For transfected cells nuclear and cytoplasmic protein extraction was carried out according to the manufacture's protocol (Thermo Scientific^™^ NE-PER^™^ Nuclear and Cytoplasmic Extraction Kit) and protein content was measured by Bradford assay. Required amount of proteins were loaded onto 10% SDS PAGE gels and subjected to immunoblotting blotting. Blocking was done in TBST with 5% non-fat dry milk (TBST-milk) or 5% BSA (BSA-TBST) for 1 h followed by 4°C overnight incubation of primary antibody SETD6 (1:1000) (HPA041481, Prestige Antibodies, Sigma-Aldrich), DDK (Flag) (1:1000), (Origene-TA50011), NF-κB p65 (SC-7151, Santa Cruz Biotechnology, Inc.), IκBα (SC-847, Santa Cruz Biotechnology, Inc.) (1:1000), phospho p65 (S36) (3033S, Cell Signaling Technology) (1:1000), IKKα (SC-7218, Santa Cruz Biotechnology, Inc.) (1:1000), IKKβ (61234, Gentetex) (1:1000), β-actin (Sigma, a1978) (1:5000), Lamin B1 (ab16048, Abcam) (1:10,000), β Tubulin Antibody (sc-9104, Santa Cruz Biotechnology, Inc.) (1:1000).

### Dual luciferase reporter assay

25,000 cells were plated in 24 well plates and co- transfection was carried out with 1 μg pCAG or pCAG-Flag SETD6 wt plasmid and 1 μg NFκB-luciferase plasmid (pGL3-NF-κB luc) together with Renilla plasmid (10 ng). Cells were lysed with 100 μl of 1X passive lysis buffer followed by freeze thaw cycles to ensure complete lysing of cells. Cells were then scraped by a scraper and collected in Eppendorf tubes and dual luciferase assay was performed according to the manufacturer's protocol (E1910, Dual-Luciferase^®^ Reporter Assay, and Promega). Samples were measured in triplicates.

### Graphs and statistical analysis

Statistical significance among two different groups was determined by student's *t* test and *P* values < 0.05 was considered significant. GraphPad Prism (GraphPad Software, Inc. La Jolla CA) was used for statistical analysis and quantitative representation of experimental data.

## SUPPLEMENTARY MATERIALS FIGURES


