# Supplementary material for: SETD6 regulates NF-κB signaling in urothelial cell survival: Implications for bladder cancer
Source: Oncotarget. 2017 Jan 19;8(9):15114–25. doi: 10.18632/oncotarget.14750 (PMC5362471; doi:10.18632/oncotarget.14750)
Supplement: Supplementary file 1 [file oncotarget-08-15114-s001.pdf]

SETD6 regulates NF-κB signaling in urothelial cell survival: Implications for bladder cancer

Supplementary Materials

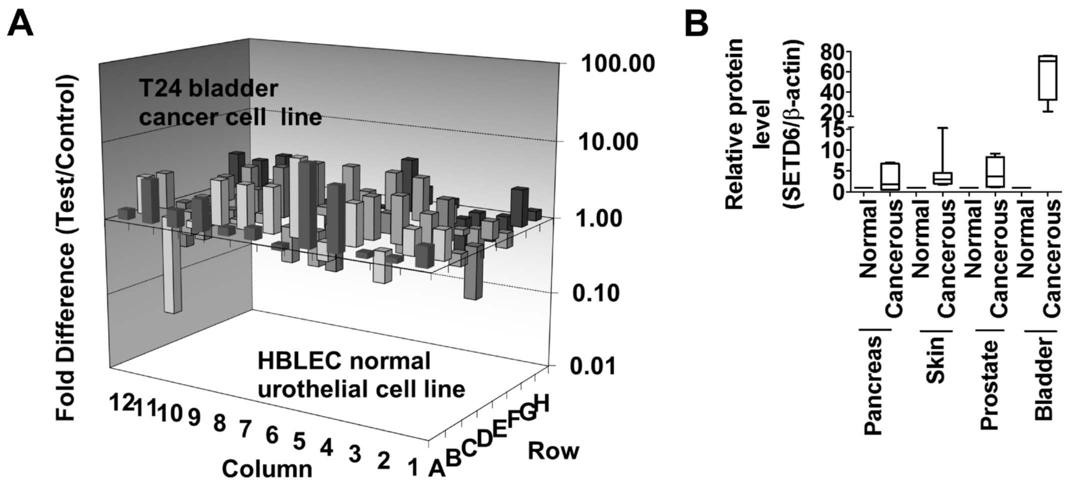

Supplementary Figure 1: Deregulation of SETD6 in cancer cells. (**A**) Representative graph of the mRNA expression of 84 genes involved in the regulation of chromatin structure and gene expression. Comparative analysis by chromatin array was made between normal urothelial cell line (HBLEC) and bladder cancer cell line (T24). The experiment was repeated twice. (**B**) Quantitative comparison of protein level of SETD6 in pancreas, skin, prostate and bladder cancers.

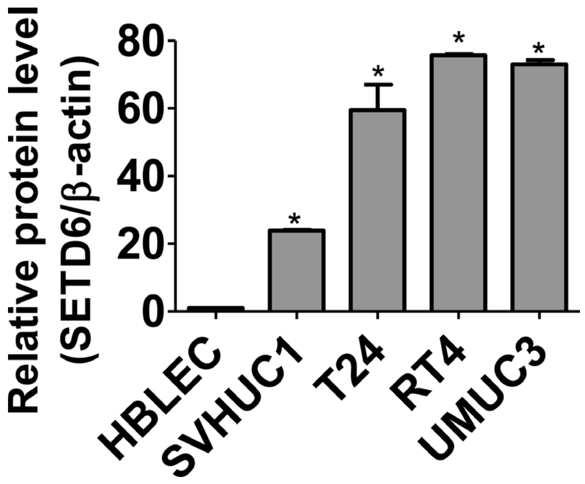

Supplementary Figure 2: Increased level of SETD6 in bladder cancer cell lines. Quantification of level of SETD6 protein in cancer and normal urothelial cell lines using western blotting. $p \leq 0.05$ was considered to be statistically significant (*) and the experiment was repeated twice.

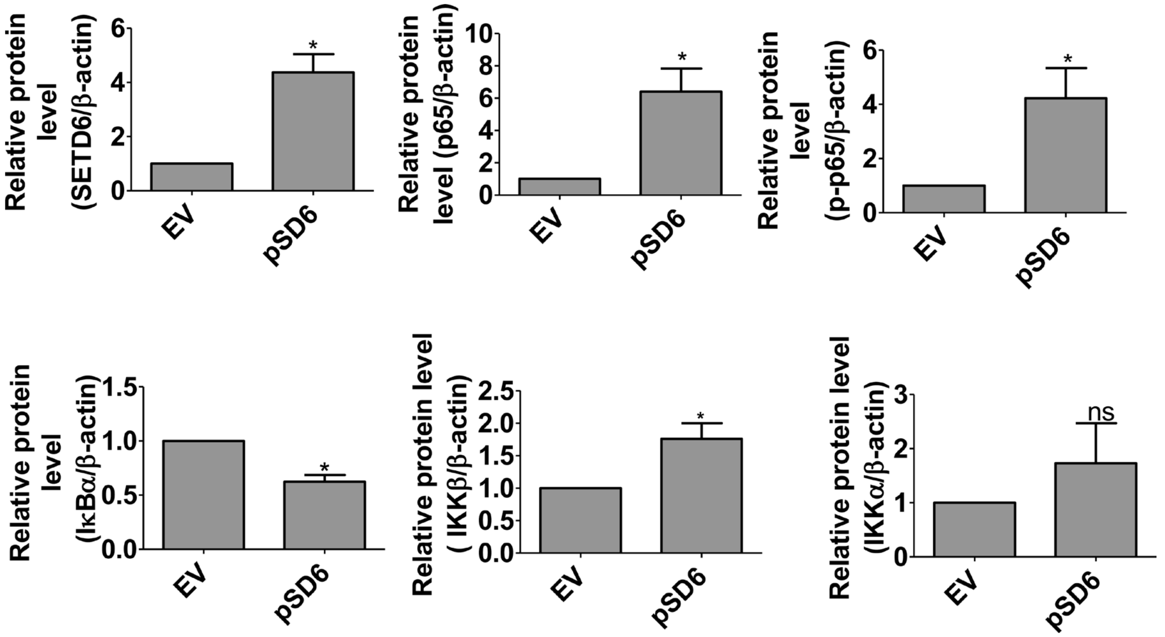

Supplementary Figure 3: Wild type SETD6 induces canonical NF-κB signaling. Quantification of the blots showing the effect of SETD6 OE on p65 and other members of the NF-κB family. $p \leq 0.05$ was considered to be statistically significant (*) and the experiment was repeated thrice. EV (empty vector): pCAG; pSD6: pCAG Flag -SETD6.

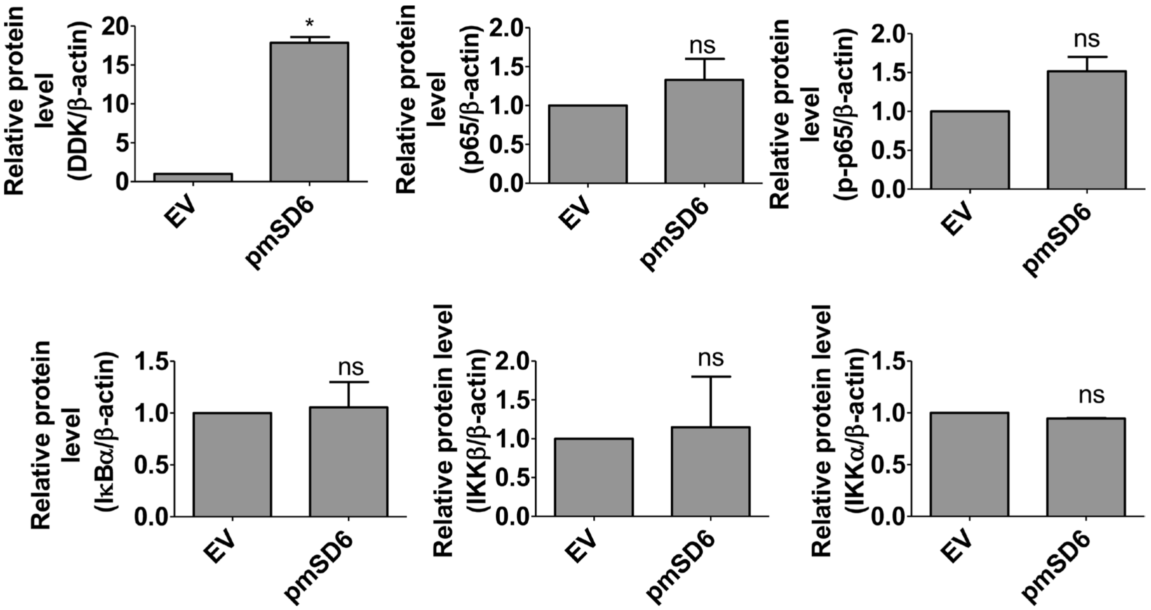

Supplementary Figure 4: Mutant SETD6 fails to induce canonical NF-κB signaling. Quantification of the blots showing the effect of mutant (mut) SETD6 overexpression on p65 and other members of the NF-κB family. $p \leq 0.05$ was considered to be statistically significant (*) and the experiment was repeated twice. EV (empty vector): pCAG; pmSD6: pCAG-Flag mutant SETD6 (Y285A).
